# Molecular Tuning of Filamin A Activities in the Context of Adhesion and Migration

**DOI:** 10.3389/fcell.2020.591323

**Published:** 2020-11-20

**Authors:** Isabelle Lamsoul, Loïc Dupré, Pierre G. Lutz

**Affiliations:** ^1^Centre de Physiopathologie de Toulouse Purpan, INSERM, CNRS, Université de Toulouse, UPS, Toulouse, France; ^2^Ludwig Boltzmann Institute for Rare and Undiagnosed Diseases, Vienna, Austria

**Keywords:** actin cytoskeleton, filamin, integrin, cell adhesion, cell migration

## Abstract

The dynamic organization of actin cytoskeleton meshworks relies on multiple actin-binding proteins endowed with distinct actin-remodeling activities. Filamin A is a large multi-domain scaffolding protein that cross-links actin filaments with orthogonal orientation in response to various stimuli. As such it plays key roles in the modulation of cell shape, cell motility, and differentiation throughout development and adult life. The essentiality and complexity of Filamin A is highlighted by mutations that lead to a variety of severe human disorders affecting multiple organs. One of the most conserved activity of Filamin A is to bridge the actin cytoskeleton to integrins, thereby maintaining the later in an inactive state. We here review the numerous mechanisms cells have developed to adjust Filamin A content and activity and focus on the function of Filamin A as a gatekeeper to integrin activation and associated adhesion and motility.

## Introduction

The filamin protein family is represented in nearly all Metazoa. Phylogenetically, the filamin genes diverge from a common single ancestral gene between chordate invertebrate and vertebrate lineages. Filamins comprise a N-terminal actin-binding domain (ABD) composed of two actin-binding calponin homology (CH) domains followed by immunoglobulin like repeats (IgFLN) of high sequence similarity (van der Flier and Sonnenberg, 2001). All C-terminal filamin repeats of filamins characterized so far have the property to homodimerize. The number of filamin repeats differs substantially in invertebrates but is almost constant in vertebrates (Light et al., 2012). The vertebrate genomes contain three filamins, Filamin A, B and C, with an intraspecies sequence identity of over 64% (Kesner et al., 2010). Filamins A and B are ubiquitously expressed, whereas Filamin C is expressed in smooth and striated muscles. Filamins A and B are localized to the cortex and stress fibers, whereas Filamin C is localized to the sarcomeric Z-line complex (van der Ven et al., 2000; Sheen et al., 2002). Disease-associated mutations and knockout mouse models suggest that Filamins A and B are critical for various aspects of skeletal, vasculature, cardio, and cerebral development (Fox et al., 1998; Sheen et al., 2002; Robertson et al., 2003; Krakow et al., 2004; Farrington-Rock et al., 2006, 2008; Feng et al., 2006; Hart et al., 2006; Lu et al., 2007; Zhou et al., 2007; Metais et al., 2018; Yamak et al., 2020), whereas Filamin C is essential for skeletal muscle and heart development (Goetsch et al., 2005; Vorgerd et al., 2005; Dalkilic et al., 2006; Duff et al., 2011; Zhou et al., 2020).

## Filamin A, a Hub for Multiple Binding Partners

**FIGURE 1:**
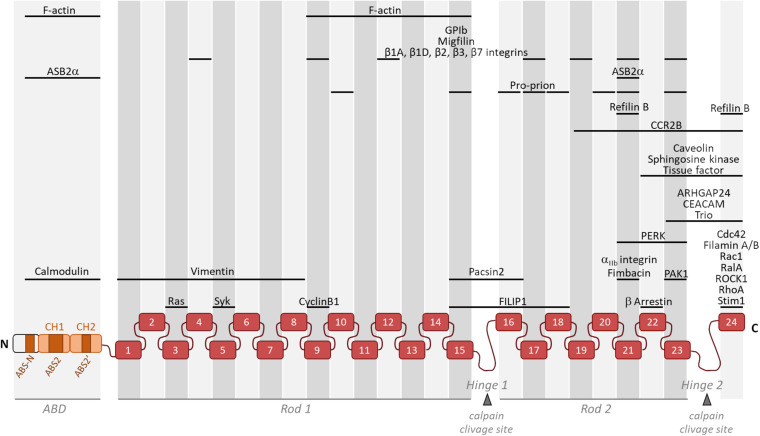
Schematic representation of monomeric Filamin A illustrating its general structure and the binding location of partners involved in actin cytoskeleton organization and/or cell motility. Filamin A consists of an actin-binding domain (ABD) composed of two actin-binding calponin homology (CH) domains followed by Ig-like repeats (1–24). Two intervening calpain-sensitive hinge domains separate the 24 Ig-like repeats into two rod domains. The three ABS (ABS-N, ABS2, and ABS2′) are indicated. Partners involved in actin cytoskeleton organization and/or cell motility but with unknown Filamin A binding domain(s) (14-3-3, ELP1, FILIP1L, p190RhoGAP, and Lck) are not represented.

Filamin A interacts with about a hundred binding-partners, many of which being involved in the regulation of signaling pathways converging toward actin cytoskeleton organization (Figure 1). Filamin A has a dual role in controlling the architecture and the mechanics of the actin cytoskeleton. Filamin A is an actin-binding and cross-linking protein whose primary function is to organize the actin cytoskeleton in orthogonal filament arrays (Nakamura et al., 2007). Importantly, the mechanic properties of this filamentous actin (F-actin) network is dependent on Filamin A concentration (Tseng et al., 2004; Gardel et al., 2006; Esue et al., 2009). At high Filamin A concentration, tighter F-actin bundles are observed and the F-actin network undergoes stress stiffening under applied forces (Schmoller et al., 2009). In contrast, at lower relative Filamin A cross-link concentrations, the F-actin cytoskeleton is more dynamic and can soften in response to stress (Tseng et al., 2004). Furthermore, the non-linear elasticity of the actin network is attributed to the flexibility of Filamin A (Kasza et al., 2009; Schmoller et al., 2009). Filamin A also localizes to points of intersection between stress fibers and cortical actin where it plays a role in the isotropic redistribution of applied forces to focal adhesions (Kumar et al., 2019). Three actin-binding sites (ABS) within the Filamin A ABD were recently identified (Figure 1; Iwamoto et al., 2018). The first one, ABS-N, located at the N-ter of the CH domain 1 contributes to F-actin binding while the two others, ABS2 and ABS2′, facilitate binding in the groove between adjacent actin subunits (Iwamoto et al., 2018). While the ABD is necessary and sufficient for F-actin binding (Razinia et al., 2011), a domain within a Filamin A fragment encompassing filamin repeats 9 to 15 is necessary for high avidity F-actin binding (Nakamura et al., 2007).

In mammals, the ABD of filamins is followed by 24 filamin repeats interrupted by two hinge regions often referred as rod 1 and 2, one between repeats 15 and 16 and another between repeats 23 and 24 (Figure 1). Filamin A domains can be divided into four subgroups (A, B, C, and D) based on amino acid similarities (Ithychanda et al., 2009b). Filamin A repeats of subgroup A (4, 9, 12, 17, 19, 21, and 23) interact with a set of biologically important ligands including platelet receptor glycoprotein Ibα (GPIbα) (Nakamura et al., 2006), migfilin (Lad et al., 2008; Ithychanda et al., 2009a, b), Cystic Fibrosis Transmembrane conductance Receptor (CFTR) (Smith et al., 2010), FilGAP (Nakamura et al., 2009), Pro-prion (Li et al., 2010), Ankyrin repeat containing protein with a SOCS box 2 alpha (ASB2α) (Lamsoul et al., 2011) and β chains of integrins (Kiema et al., 2006; Takala et al., 2008). All subgroup A Filamin A repeats and their binding partners have similar mode of interaction. Indeed, the CD face of Filamin A repeats represents a common interface for Filamin A-ligand interaction (Lad et al., 2007; Heikkinen et al., 2009). Interestingly, Filamin A repeats can also engage in intramolecular contacts (IgFLNa16-17, IgFLNa18-19, and IgFLNa20-21) that may become disrupted by binding of one of the repeats to the integrin β cytoplasmic tail or by mechanical forces (Lad et al., 2007; Heikkinen et al., 2009). Smoothelins A and B, as well as fimbacin can bind to the cryptic CD cleft of Filamin A repeat 21 exposed in mechanically activated Filamin A (Wang and Nakamura, 2019a, b). Filamin A can also interact with small GTPases of the Rho family, Rac, Rho, cdc42, and RalA (Ohta et al., 1999; Bellanger et al., 2000; Vadlamudi et al., 2002; Ueda et al., 2003) and with proteins upstream and downstream of the GTPases (Ohta et al., 2006; Nakamura et al., 2009) known to regulate cytoskeletal dynamics and cell protrusions. In addition, RhoA activity is downregulated through interactions between Filamin A and αIIbβ3 integrins and is critical to proplatelet formation (Donada et al., 2019).

Filamin A is also localized into the nucleus where it plays roles in DNA repair through interaction with BRCA1 and BRCA2 (Yue et al., 2009; Velkova et al., 2010), as well as transcription through interaction with transcription factors such as the androgen receptor (Loy et al., 2003; McGrath et al., 2013; Savoy et al., 2015), Smads (Sasaki et al., 2001) or PEBP2β/CBFβ (Yoshida et al., 2005). Filamin A also associates with the MKL1 transcriptional co-activator, stimulating the activity of the Serum Responsive Factor (SRF) transcription factor and cell migration (Kircher et al., 2015).

How Filamin A integrates the signals triggered by its multiple binding partners and whether such complex molecular interactions might be tuned differentially in different cell types remain key open questions. Nevertheless, the essentiality of Filamin A is highlighted by variants in the gene *FLNA* that lead to 10 distinct genetic syndromes affecting a wide diversity of organs (Wade et al., 2020). Importantly, pathogenic variants of *FLNA* could contribute to aberrant cytoskeletal regulation leading either to loss-of-function or gain-of function disorders. Indeed, variants are found in the two CH domains of the ABD, CHD1 and 2, in periventricular nodular heterotopia (PH) and otopalatodigital (OPD) syndromes type 1 and 2, respectively. Variants within CHD1 are likely to disrupt Filamin A interaction with actin (Iwamoto et al., 2018) whereas variants within CHD2 are likely to constitutively expose the CHD1 ABS to ligand (Clark et al., 2009). Interestingly, a mutation in *FLNA* in a male patient with PH and congenital intestinal pseudoobstruction potentiates αIIbβ3 integrin activation likely through less binding of mutant Filamin A to β3 integrin and facilitated recruitment of Talin by the β3 subunit (Berrou et al., 2017). The relationship between other *FLNA* pathogenic variants and Filamin A functions is less understood and remains to be investigated.

## Filamin A, a Negative Regulator of Integrins

Because the filamin domains involved in binding actin and integrins have the highest content of ancestral residues of any domains (Kesner et al., 2010), integrins are considered among the most important interaction partners of filamins. Integrins are heterodimeric transmembrane receptors formed by α and β subunits. These are single spanning membrane proteins with a large extracellular ectodomain and a short intracellular cytoplasmic tail. Integrins mediate cell to extracellular matrix and cell to cell contacts and integrate external cues to the actin cytoskeleton and signaling pathways (Legate and Fassler, 2009; Humphries et al., 2019; Kechagia et al., 2019). Interactions between integrins and their extracellular ligands are tightly regulated thanks to integrin activators and integrin inhibitors. Importantly, switching integrins between inactive and active conformations is crucial for integrin functions (Bouvard et al., 2013). Integrin activation has largely been documented (Kim et al., 2011; Sun et al., 2019). This process is regulated via either extracellular ligands (outside-in activation) or intracellular binding partners (inside-out activation). Integrin-inactivating proteins such as integrin cytoplasmic domain-associated protein 1 (ICAP1), SHARPIN (SHANK-associated RH domain-interacting protein) and filamins are required for integrin inactivation in different settings (Calderwood et al., 2001; Bouvard et al., 2003; Rantala et al., 2011). The physiological relevance of integrin-inactivating proteins is crucial for integrin function as exemplified by the phenotypes of mice lacking integrin inactivators (Bouvard et al., 2013). Integrin inactivators either stabilize the inactive state of integrins or promote integrin deactivation during cyclical cell-adhesion processes such as migration. Indeed, a substantial proportion of cell surface integrins is in a resting state (Arjonen et al., 2012). Inactive integrins are in a closed conformation, in which the binding of both extracellular ligand and intracellular activators is repressed. In human, there are several integrin β subunits that bind filamins (Kiema et al., 2006; Ithychanda et al., 2009b).

Filamin A is a major gatekeeper to integrin activation. Since the discovery of Filamin A as a binding partner of the β2 integrin subunit 25-years ago (Sharma et al., 1995) and the first evidence that increased Filamin A-β2 integrin interactions restrict cell migration (Calderwood et al., 2001; Bouvard et al., 2003; Rantala et al., 2011), several modes of action of Filamin A as an integrin inactivator have been proposed. They depend on the identity of the integrin α and β chains or could be specific to only a subset of integrin αβ heterodimers. First, binding of Filamin A domains of subgroup A to the C terminus of the integrin β tail (β1, β2, β3, or β7) results in direct competition with talin binding by occupying an overlapping binding site (Kiema et al., 2006; Ithychanda et al., 2009b). Second, Filamin A forms a ternary complex engaging the cytoplasmic tails of both integrin αIIb and β3, thereby stabilizing the inner-membrane clasp and competing with talin recruitment to the β subunit cytoplasmic tail by binding both the C-terminal and membrane-proximal regions of the β3 tail (Liu et al., 2015). These two modes of action of Filamin A restrain the integrin in a resting state. Interestingly, domains within functionally important binding interfaces of both filamin repeats and integrin subunits have diverged in critical residues, indicating that filamin isoforms may bind and regulate integrin αβ heterodimers differentially. Indeed, Kesner et al. (2010) described the substitution in the β strand C of the filamin repeat 21, an ancestral Ser/Thr in Filamins B and C changed to an Ala at residue 2272 in Filamin A during the mammalian period. Furthermore, β1 and β7 integrins have ideally positioned hydrophobic amino acids to bind Filamin tighter than β2 and β3 integrins (Ithychanda et al., 2009b). Furthermore, some of the key residues in the αIIb subunit that are important for interaction with filamin via their side chains, are not conserved in all integrin α subunits, reinforcing the notion that filamins bind integrins differentially.

Because several Filamin A repeats can bind the cytoplasmic tails of β integrins and have the ability to clasp αIIb and β3 cytoplasmic tails, it seems plausible that they can bind simultaneously, and such interactions may promote clustering of inactive integrins (Ithychanda et al., 2009b; Liu et al., 2015). Although the biological significance of these Filamin A clutches remains to be establish, it is tempting to speculate that upon Filamin A removal, pre-clustered integrins would become engaged by multivalent ECM and thereby activated.

## Multiple Regulatory Mechanisms Controling Filamin A Activities and Levels

Integrin activation can be achieved through the binding of proteins to Filamin A. Indeed, migfilin can bind Filamin A with a high affinity, uncoupling the Filamin A-integrin link, sequestering Filamin A away from the β integrin cytoplasmic tail and thus counteracting Filamin A-mediated integrin inactivation (Lad et al., 2008; Ithychanda et al., 2009a; Das et al., 2011). This allows the binding of integrin activators, talin and kindlins, to the β integrin cytoplasmic tail, leading to inside-out integrin activation (Tadokoro et al., 2003; Tu et al., 2003; Kiema et al., 2006; Wegener et al., 2007; Moser et al., 2008). Internally generated and externally imposed mechanical forces can also regulate Filamin A interaction with partners by triggering conformational changes that expose otherwise masked partner-binding site, thereby leading to integrin activation (Ehrlicher et al., 2011; Nakamura et al., 2014). Although evidence for the mechanosensing function of Filamin A in Drosophila oogenesis has been provided, its precise role in cell differentiation and morphogenesis in mammals is still lacking (Razinia et al., 2012; Huelsmann et al., 2016). Filamin A is also regulated by phosphorylation. Several kinases such as protein kinase C (Kawamoto and Hidaka, 1984), ribosomal S6 kinase (Ohta and Hartwig, 1996; Woo et al., 2004), p21-activated kinase 1 (PAK1) (Vadlamudi et al., 2002; Hammer et al., 2013), the cyclic adenosine monophosphate (cAMP)-dependent protein kinase A (Jay et al., 2004), Akt (Li et al., 2015), mTOR2 (Chantaravisoot et al., 2015; Sato et al., 2016) and the serine/threonine kinase Ndr2 (Waldt et al., 2018) phosphorylate Filamin A on serine 2152. This phosphorylation event positively regulates cell migration (Woo et al., 2004; Hammer et al., 2013; Li et al., 2015; Sato et al., 2016). Activation of receptor tyrosine kinases was shown to trigger cell rounding and integrin inactivation via increased Filamin A phosphorylation (Vial and McKeown-Longo, 2012; Mai et al., 2014). In addition, G Protein-Coupled Receptors that directly bind Filamin A can also promote Filamin A phosphorylation (Tirupula et al., 2015). Filamin A phosphorylation by the cAMP-dependent protein kinase protects Filamin A against proteolysis by calpains (Chen and Stracher, 1989). Phosphorylation of β2 integrins impairs Filamin A binding, allowing the binding of the 14-3-3 protein to the β2 subunit and adhesion of Jurkat T cells to ICAM-1 (Takala et al., 2008).

**FIGURE 2:**
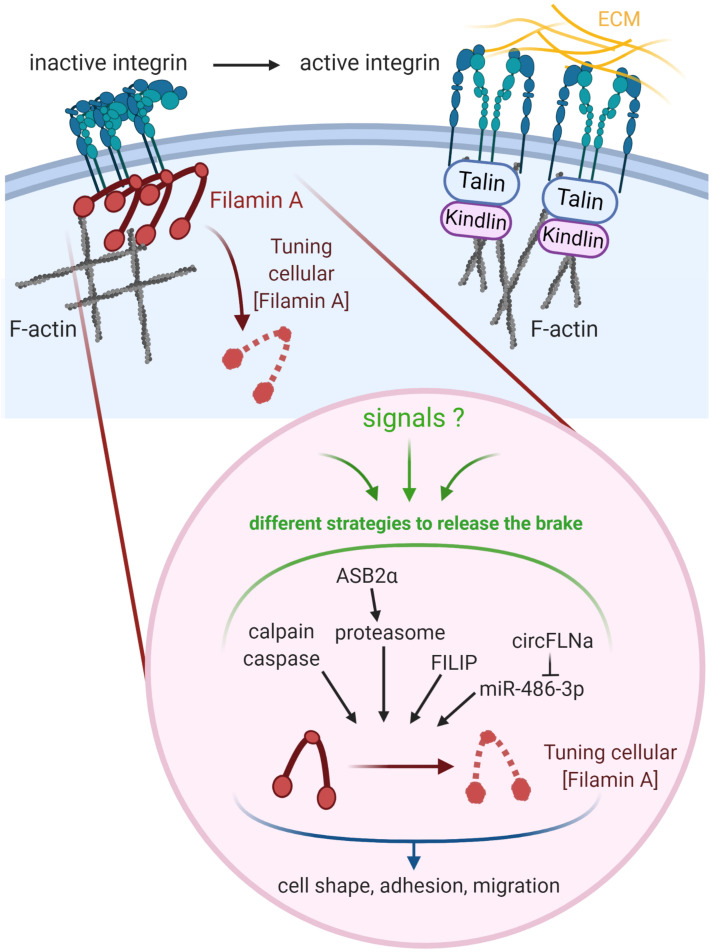
Tuning the cellular concentration of Filamin A represents a pivotal mechanism to regulate integrin-dependent adhesion and migration. Regulation of integrins involves both integrin inhibitors (e.g., Filamin A) and integrin activators (e.g., Talin, Kindlin). Filamin A protein is regulated through cleavages by calpain and caspase, ASB2α-mediated degradation by the proteasome, and interaction with FILIP. Filamin A mRNA is down-regulated by miR-486-3p which is itself sponged by circFLNA. Created with BioRender.com.

Tuning the cellular concentration of Filamin A represents another level of regulation expected to impact integrin activation, although this has not been formally demonstrated yet (Figure 2). Filamins are regulated by proteolysis, which provides an irreversible regulatory mechanism for processes requiring Filamin removal. Filamin A is cleaved by calpain and caspase at the two hinge regions, producing a 170 kDa protein encompassing the ABD and Filamin A repeats 1 to 15 and a 110 kDa protein that is further cleaved to generate a 90 kDa fragment containing repeats 16 to 23 (Figure 1) (Gorlin et al., 1990; Browne et al., 2000). Filamins A and B are also regulated by proteasomal degradation which represents a fast, reversible, localized and selective regulatory mechanism that allows cells to acutely adapt or fine-tune cellular processes. Surprisingly, only few proteins linked to cytoskeleton dynamics, cell adhesion and migration have been shown to be regulated by this proteolysis pathway in non-muscle cells (Schaefer et al., 2012). Of interest, control of the cellular concentration of Filamins A through ubiquitin-mediated protein degradation represents a seminal example of proteasomal degradation of an actin-binding and -crosslinking protein. Using several molecular and cellular biology approaches, we and others demonstrated that the ASB2α E3 ubiquitin ligase (E3) triggers ubiquitylation and proteasomal degradation of Filamins A and B (Heuze et al., 2008; Burande et al., 2009; Lamsoul et al., 2013; Razinia et al., 2013; Sakane et al., 2013; Spinner et al., 2015; Metais et al., 2018). ASB2α is the specificity subunit of a multimeric E3 of the Cullin 5-RING Ligase family involved in the recruitment of proteins to be ubiquitylated (Lamsoul et al., 2016). By degrading Filamins A and B, ASB2α regulates cell spreading, adhesion and cell migration (Heuze et al., 2008; Baldassarre et al., 2009; Lamsoul et al., 2011, 2013; Spinner et al., 2015). Furthermore, our recent results support a model of cardiac cell differentiation that relies on a key role for ASB2α in remodeling the actin cytoskeleton through induced-degradation of Filamin A (Metais et al., 2018). Indeed, the timely controlled removal of Filamin A ensures critical functions in differentiating cardiac muscle cells suggesting that Filamin A degradation is necessary to modify the actin cytoskeleton organization and properties in order to build the sarcomere, and thus for heartbeats. In addition, the Filamin A interacting protein (FILIP) interacts with Filamin A and induces its degradation with impacts on the mode of neuron migration (Nagano et al., 2004; Sato and Nagano, 2005). More recently, Filamin A expression was shown to be regulated by a microRNA (miR) and a circular RNA (circRNA). Indeed, miR-486-3p can bind Filamin A 3′UTR thereby reducing Filamin A expression while circFLNA sponges miR-486-3p resulting in increased Filamin A expression (Wang et al., 2019). Important questions remain: Does Filamin A degradation directly translate into increased integrin activation? What is the biological relevance of variable Filamin A levels in different cell subtypes or at discrete stages of cell differentiation? Why cells have evolved so many different mechanisms to regulate Filamin A activity and to up-regulate or down-regulate Filamin A concentration?

## Filamin A in Cell Adhesion and Migration

The first evidence for a role of Filamin A in cell motility was provided in 1992 (Cunningham et al., 1992). Indeed, at the cellular level, Filamin A deficiency in a human melanoma cell line promotes plasma membrane blebbing and causes loss of motility. The role of Filamin A in migration was further supported by the finding that nonsense mutations in the Filamin A gene are associated with the neuronal migration disorder periventricular heterotopia (Fox et al., 1998). However, the role of Filamin A in cell motility is more complex. By providing a physical link between integrins and the actin cytoskeleton and by negatively regulating integrins, Filamin A exerts key roles in regulating positively or negatively cell adhesion and migration according to cell types and/or conditions. Furthermore, Filamin A binding partners may vary according to cell types and/or in response to microenvironment cues such as extracellular matrix components, chemokines or shear flow. This is likely to influence cell adhesion and migration. While the loss of Filamins A or B alone has no effect on cell motility, loss of both filamins following knockdown/knockout or ASB2α-mediated degradation have highlighted the role of filamins in different aspects of cell motility (Heuze et al., 2008; Baldassarre et al., 2009; Lynch et al., 2011; Lamsoul et al., 2013; Spinner et al., 2015). It is tempting to speculate that the functions of Filamin A in many cell types may have been missed in assays using Filamin A single knockout/knockdown cells because of compensation by Filamin B (Sheen et al., 2002; Baldassarre et al., 2009). Filamin-depleted cells exhibit impaired cell spreading (Heuze et al., 2008; Kim et al., 2008; Baldassarre et al., 2009; Lynch et al., 2011). In addition, increased adhesion of Filamin A-depleted neutrophils has been described (Sun et al., 2013). Furthermore, Filamin A knockdown or ASB2α-mediated Filamin A degradation enhances adhesion of myeloid leukemia cells to fibronectin (Lamsoul et al., 2011). In contrast, Roth et al. found that Filamin A was dispensable for adhesion of differentiated HL-60 cells (Roth et al., 2017). However, Filamin A depleted primary murine neutrophils display increased spreading on and higher adhesion in shear-free conditions to β2 integrin ligands, indicating that Filamin A is a negative regulator of β2 integrin adhesion in neutrophils (Uotila et al., 2017). Although Filamin A negatively regulates β2 integrin adhesion in Jurkat T cells, its absence leads to a reduction of primary T cell adhesion to integrin ligands under conditions of shear flow and to a reduced trafficking into lymph nodes and sites of inflammation (Moser et al., 2009; Savinko et al., 2018). Interestingly, Filamin A and vimentin can cooperate to regulate integrin-mediated cell spreading and cell adhesion (Kim et al., 2010a, b).

Filamins A and B depleted cells exhibit impaired initiation of migration of fibrosarcoma HT1080 cells (Baldassarre et al., 2009). Filamin A silencing increases cell adhesion and decreased migration of the bronchial carcinoid H727 cells (Vitali et al., 2017). In contrast, silencing of Filamin A inhibits Snail-induced adhesion and increases migration of colon adenocarcinoma HT29 cells (Wieczorek et al., 2017). In accordance with these results, Filamin A is required to mediate SST2 effects on adhesion and migration of the pancreatic endocrine QGP1 cells (Vitali et al., 2016). Filamin A also positively regulates directional migration of bone osteosarcoma U-2 OS cells and mouse embryonic fibroblasts by suppressing Rac 1 activity downstream of β1 integrins (Jacquemet et al., 2013). Knockdown of Filamins A and B in fibrosarcoma cells was also shown to augment matrix metalloproteinase activity increasing their invasive potential (Baldassarre et al., 2012). These results are in agreement with the fact that ASB2α regulates immature dendritic cell migration by promoting extracellular matrix proteolysis (Lamsoul et al., 2013). Conversely, Filamin A stabilizes podosomes in macrophages and is required for their mesenchymal but not for their amoeboid migration (Guiet et al., 2012). In addition, in the absence of Filamin A, macrophages display impaired migration associated with reduced atherosclerosis in mice (Bandaru et al., 2019). Several evidences indicate that Filamin A regulates the intracellular trafficking of β1 integrins (Meyer et al., 1998; Kim et al., 2010b). This is likely to affect β1 integrin-dependent processes. On the basis of these scattered observations, it is clear that we still miss today a unified view of the roles of Filamin A in cell adhesion and migration.

## Concluding Remarks

As reviewed here, the timely proteolysis and/or removal of Filamin A have emerged as pivotal mechanisms to regulate its cellular concentration and integrin-dependent adhesion and migration. When integrating the knowledge gained about the function of Filamin A beyond its integrin regulation role, one is tempted to speculate that this key protein at the interface between multiple receptors, signaling pathways and the actin cytoskeleton exerts different and specific cellular functions in response to a wide-range of environmental cues. As exemplified by the wide spectrum of developmental malformations and diseases caused by mutations in its gene, Filamin A indeed stands out as a major molecular player in different biological processes. In this context, it will be particularly interesting to further investigate how the multiple mechanisms able to adjust Filamin A concentration and activity contribute to its function in different cellular and physiological settings.

## Author Contributions

All authors contributed to both the review conceptualization and the writing process.

## Conflict of Interest

The authors declare that the research was conducted in the absence of any commercial or financial relationships that could be construed as a potential conflict of interest.
